# Relating Lying Behavior With Climate, Body Condition Score, and Milk Production in Dairy Cows

**DOI:** 10.3389/fvets.2020.565415

**Published:** 2020-11-05

**Authors:** Daniela Lovarelli, Alberto Tamburini, Gabriele Mattachini, Maddalena Zucali, Elisabetta Riva, Giorgio Provolo, Marcella Guarino

**Affiliations:** ^1^Department of Environmental Science and Policy, Università degli Studi di Milano, Milano, Italy; ^2^Department of Agricultural and Environmental Sciences, Università degli Studi di Milano, Milano, Italy

**Keywords:** lying, lactation stage, temperature-humidity index, primiparous dairy cattle, precision dairy farming

## Abstract

Attention on animal behavior and welfare has been increasing. Scientific knowledge about the effect of behavior and welfare on animals' production augmented and made clear the need of improving their living conditions. Among the variables to monitor in dairy cattle farming, lying time represents a signal for health and welfare status as well as for milk production. The aim of this study is to identify the relationship among the lying behavior of dairy cows and milk production, body condition score (BCS), weather variables, and the temperature–humidity index (THI) in the barn from a dairy farm located in Northern Italy. One-year data were collected on this farm with sensors that allowed monitoring of the environmental conditions in the barn and the activity of primiparous lactating cows. Principal components analysis (PCA), factor analysis (FA), generalized linear model select (GLMSelect), and logistic analysis (LA) were carried out to get the relationships among variables. Among the main results, it emerges that the effect of weather parameters is quite restrained, except for THI > 70, which negatively affects the lying time. In addition, the most productive cows are found to lie down more than the less productive ones, and the parameters of milk production, lying time, and BCS are found to be linked by a similar trend.

## Introduction

In recent years, attention on animal behavior and welfare has increased considerably (1, 2). There are several reasons for this higher consideration. Firstly, scientific knowledge has increased, demonstrating the need to make cows live in a safe and healthy environment to improve their welfare conditions and the related milk production (2, 3). Secondly, the market and the consumers have been affecting production patterns because of their increased consciousness about the living conditions of animals (4). Finally, technological progress is giving the possibility to stakeholders, mainly farmers, policymakers, and consumers, to obtain a big amount of data on production aspects and on the relation among the different parameters. This opportunity reflects on the farmers' capability to monitor continuously the reared animals, get advantages from the animal-by-animal control, and introduce prompt interventions when necessary (3, 5–7). Technology, wireless connectivity, sensors, and monitoring tools are adopted in this analysis framework, recognized as precision livestock farming (PLF). PLF is defined as a multidisciplinary science that puts its basis on the collaboration of several expert figures in order to manage animals individually, continuously, and in real time on multiple aspects of health, welfare, production, and reproduction (6). This supports farmers who have difficulties in visually monitoring animals in large herds without technological support.

In addition, with the indispensable need of increasing aspects such as production efficiency, animals' health and welfare, and workers' health and welfare, PLF increases in importance (5, 8). In this context, the sustainability of livestock productions can also improve (2, 3, 7).

Among the aspects affecting production, welfare, and health issues, heat stress is not a negligible problem (9, 10), causing both direct and indirect lifelong side effects to cows. Therefore, numerous studies have been carried out on this aspect and on its different facets: with respect to milk production (11), to reproduction efficiency (11), to activity (12), and to the barn environment (9). In any case, monitoring animals' behavior is a fundamental aspect. As widely known, lying behavior is a symptom of lack of welfare and/or healthy animals. In the latter case, cows lie down on average about 9–14 h/day (13), a range out of which anomalies and side effects arise. In particular, when cows lie down, they ruminate, hence affecting milk production level. On the contrary, when they lie down more or less than the range, health problems, bad barn engineering, or heat stress are the most common causes (14). Therefore, modeling their lying behavior has undoubted importance for the farmer.

Among the factors that affect cow behavior, the temperature–humidity index (THI) has been widely used to assess the effect of environmental conditions and mainly of heat stress (11, 15). Several authors reported an increase of the standing time with the increase of THI and the consequent reduction in milk production (13, 16–19).

In this study, lactating cows in a commercial farm were analyzed with regard to the aspects that affect the lying behavior of primiparous dairy cattle with the aim of identifying the variables that have influence on the lying behavior and getting relations among them. The monitoring was also aimed to identify if a seasonal trend in the lying time of dairy cattle existed in the farm.

## Materials and Methods

### Livestock Farm

The commercial dairy farm “A. Menozzi” is analyzed in this study. The farm is located in Landriano (PV), in Northern Italy. In total, about 92 Italian Holstein lactating cows are present. Housing consists of free-stall pens in a loose-housing layout; 130 cubicles with synthetic mattresses and 106 feeding places are present, split into two boxes, one for fresh cows (i.e., cows at the beginning of the lactation period) and one for late lactating cows (i.e., cows in the last phase of the lactation period). The total mixed ration (TMR) is delivered daily in the morning, and cows are milked twice a day. As cooling system, fans and sprinkler are installed in the feeding area, while destratifiers are present in the resting area.

In this study, 20 primiparous cows were monitored for 1 year to collect data about the environmental barn conditions, animals' activity, milk production, and body condition score (BCS). Their calving period ranged between October and March.

Environmental and behavioral characteristics were monitored by means of sensors installed in the barn (for environmental aspects) and on the hind leg of animals (for their behavior) as described hereby (**section Sensors**). Information on daily milk production of each cow was obtained from the monitoring instrumentation available on farm for the herd management. Individual BCS was assessed for all the monitored primiparous cows at the calving stage and every week of the trial using the visual technique and classification as proposed in (20).

### Sensors

Two typologies of sensors were installed on the farm: one for the environmental aspects and the other for the behavioral ones.

#### Environmental Assessment

With the environmental data, information about climatic variables inside the barn was collected and analyzed in order to evaluate the living conditions of dairy cows. HOBO U12 Temp/RH/Light/External Data Logger (Onset Computer Corporation, Bourne, MA, USA) sensors were installed in four different positions of the barn at 2-m height. They continuously collected data during 1 year.

Temperature and relative humidity in the barn were used to quantify the THI and evaluate the possible presence of unwanted conditions, such as heat stress. THI was calculated in accordance with ASABE (16) using **equation (1)**.

(1) $$\begin{array}{l} \text{THI}=\text{T}+0.36\times {\text{T}}_{\text{dp}}+41.2 \end{array}$$

where T = dry bulb temperature (°C) and T_dp_ = dew point temperature (°C).

In addition, illuminance was considered in the data analysis as a parameter that helps characterize the season: high illuminance was obviously associated with spring and summer periods.

The number of hours per day in which THI was higher than 70 [identified as the threshold value according to (13)] was calculated to identify how prolonged is the condition of heat stress, if present. In particular, more negative effects caused by heat stress may be present when heat stress is prolonged for several hours (21–23). Therefore, the variable “THI > 70” was introduced in data processing, indicating the number of consecutive hours per day with THI > 70.

Finally, environmental data outside of the barn were collected using a meteorological station installed on the farm by the Regional Agency for the Protection of the Environment (ARPA) (24).

#### Behavioral Assessment

Accelerometers were installed on the hind leg of the primiparous lactating cows to evaluate their activity, indicating the number of hours they were lying (h/day) or standing (h/day), the number of daily lying bouts of cows (no. of bouts/day), and the duration of these lying bouts (min/day).

The adopted tools were HOBO Pendant G Data Loggers (Onset Computer Corporation, Pocasset, MA, United States) that were installed individually to record continuously the activity and, in particular, to record the leg orientation to detect the lying activity. This device recorded data at 1-min intervals for the whole monitoring period of each primiparous cow, which was 150 days. In total, the measurement lasted 1 year.

### Statistical Analysis

SAS 9.4 software (SAS, Cary, NC, United States) was used for the statistical analysis of the data collected on the farm [i.e., days in milking (DIM), milk production, BCS, lying time, number of lying bouts, bout duration, illuminance, temperature and humidity, stall temperature and humidity, wind speed, and rainfall]. The first step in data processing involved the analysis of raw data and deletion of outliers and sensors malfunctioning.

Environmental and behavioral data were averaged per day in order to get the same temporal basis with weather, milk production, and BCS. Descriptive statistics were carried out to describe the variables present in the dataset. Variable classification was done to divide the dataset into defined homogeneous groups descriptive of the farm. Classification occurred for the following:

- Lactation stage, which included splitting milk production in three groups of about 50 DIM each. “Lact. stage 1” for <50 DIM, “Lact. stage 2” for 50–100 DIM, and “Lact. stage 3” for >100 DIM;
- Standing time during the first 21 DIM, which was split into two groups, namely, “high standing time” >14 h/day and “low standing time” ≤14 h/day;
- BCS at calving, which was split into two groups, namely, “high BCS” >3.25 and “low BCS” ≤3.25; and
- Milk production level, which was built on two groups, “high milk production” >28 kg/day and “low milk production” ≤28 kg/day.

To better analyze the relation between milk production and cattle behavior, two indexes were computed for each cow and each day of the trial. These indexes aim at analyzing the efficiency of milk production in relation with the daily behavior of cows by taking into account the two main aspects describing the behavior, which are the daily lying time and the number of lying bouts per day:

The lying efficiency index (LEI) is calculated as the ratio between daily milk production and daily lying time, hence resulting in the milk produced in every hour in which cows lie down (kg/h) according to the following equation:

(2) $$\begin{array}{l} \text{LEI}=\text{Milkproduction}/\text{Lyingtime} \end{array}$$

where LEI is measure in kg/h, milk production in kg/day, and lying time in h/day.

The bout number efficiency index (BNEI) is calculated as the ratio between milk production and the number of lying bouts per day, hence resulting in the daily milk produced per bout completed by the cow (kg/no. of bouts). It is calculated according to the following equation:

(3) $$\begin{array}{l} \text{BNEI}=\text{Milkproduction}/\text{Boutnumber} \end{array}$$

where BNEI is measured in kg/no. of bouts, milk production in kg/day, and bout number in no. of bouts/day.

After this step, multivariate statistics were carried out using SAS 9.4 to get the relationship among variables in the livestock farm with respect to the lying time and to gradually deepen all the aspects that affect animal behavior. This series of analyses was carried out because the livestock is a complex ambient with many variables affecting each other.

First, principal components analysis (PCA) (Proc PRIN COMP) and factor analysis (FA) (Proc FACTOR, no rotation, and method PRINCIPAL) were carried out to show the graphs of the components and to understand the relationship between the single variables (PCA) and to understand the relationships among variables and principal components (FA). Secondly, generalized linear model select (GLMSelect) (Proc GLMSelect) and logistic analysis (LA) (Proc LOGISTIC) were conducted to further investigate the studied system. LA was done using a binary logit and Fishers' scoring as an optimization technique. Finally, cluster analysis (CA) followed, through which animals were clustered in homogeneous groups characterized by similar attributes.

## Results

### Descriptive Statistics

Table 1 reports the results on the main parameters descriptive of the studied farm. In total, 2,712 observations of 20 cows were available for the analysis.

**Table 1:** Mean, standard deviation, coefficient of variation, and minimum and maximum values for the main parameters descriptive of the study farm.

**Parameters**	**Unit**	**Mean ± SD**	**CV**	**Min**	**Max**
DIM	day	78.9 ± 44.8	56.8	1.0	194
Milk production	kg/day	27.9 ± 6.46	23.2	8.0	45.8
Lying time	h/day	10.6 ± 2.64	25.7	0.02	19.2
No. of lying bouts	no/day	8.80 ± 3.93	44.6	0.0	34.0
Bout duration	min/day	87.9 ± 46.6	52.9	0.0	448
BCS	–	3.36 ± 0.28	8.36	2.75	4.00
Illuminance	lux	762 ± 596	78.2	26.9	2,599
THI internal	–	57.0 ± 8.74	15.4	41.6	76.3
THI > 70	h/day	2.43 ± 5.17	213	0.0	24.0
THI external	–	55.7 ± 9.01	16.2	40.6	75.9
Wind speed	m/s	1.59 ± 0.75	47.1	0.22	6.23
Rainfall	mm	4.09 ± 8.59	210	0.0	49.8
LEI	kg_MILK_/h_LYING_	2.68 ± 0.83	31.1	0.68	6.63
BNEI	kg_MILK_/no. of bouts	3.66 ± 1.73	47.4	0.61	10.0

The average milk production was 27.9 ± 6.5 kg/day. Although not reported in the table, milk production achieved the highest average value during the intermediate lactation stage (Lact. stage 2, 50–100 DIM) when it was equal to 29.1 ± 6.0 kg/day. Daily milk production was lower at the beginning of the lactation stage (Lact. stage 1, <50 DIM), when the average was 25.3 ± 5.5 kg/day, while it maintained the trend of Lact. stage 2 also during Lact. stage 3 (>100 DIM), with the average production being equal to 28.9 ± 5.7 kg/day.

These primiparous cows lie down on average 10.6 ± 2.7 h/day, with about 8.8 ± 3.9 lying bouts/day and a duration of lying bouts equal to 87.9 ± 46.6 min/day. The average BCS at calving was 3.4 ± 0.3. Most of calving took place in autumn and in winter.

Regarding the environmental aspects, THI was on average 57.0 ± 8.7, although it reached values of 76.3 during summer; in addition, the duration of conditions with THI > 70 occurred on average for 2.4 ± 5.2 h/day. This result, being an average, is affected by the fact that most data were collected in cold-temperate seasons. The THI external to the barn resulted on average to a value very close to that in the barn (55.7 ± 9.0, ranging between 40.6 and 75.9). Wind and rainfall were not particularly intense, although heavy rain occurred in some cases.

The LEI and BNEI show interesting values. Every hour in which cows lie down, they produced on average 2.68 ± 8.6 kg of milk, varying in the range 0.68–6.63 kg/h; BNEI shows that in every bout, they produced on average 3.66 ± 1.73 kg of milk, varying in the range 0.61–10.0 kg/bout. This result is interesting because it helps us understand the effect of cows' behavior on milk production and that every variation in behavior can play a non-negligible role on total milk production.

### Multivariate Statistics

Because the trial period started in September and ended the next August, DIM and THI resulted in a strong relationship (*r* = 0.42, data not shown). Similarly, because BCS was measured weekly during the lactation stage of each cow, a strong relationship was found also between DIM and BCS (*r* = 0.59, data not shown). Moreover, it emerged also that during lactation, milk production increased with no reduction in production because the analysis ended around 150 DIM. As expected, THI and illuminance were well-related (*r* = 0.53, data not shown) because with the lengthening of the lactation period, the warmer and longer daylight period occurred, since for most cows the calving period was in winter. Finally, an inverse relation between number of lying bouts and their duration was found as well (*r* = 0.37, data not shown).

The PCA was carried out by including variables related to milk production, DIM, behavior (lying time and number and duration of bouts), BCS, and weather (THI, THI > 70, illuminance, wind speed, and rainfall).

**Figure 1:**
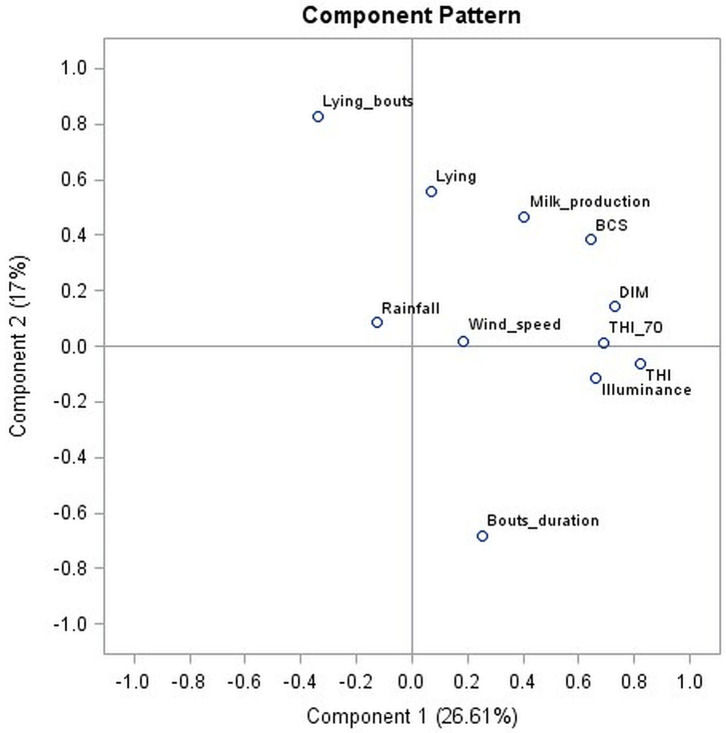
PCA for component 1 and component 2.

The first four eigenvalues identified with PCA analysis explain 60% of the data variability. Figure 1 shows these results only for components 1 and 2 (43.6% of the data variability) for simplicity.

From the results of the first two components of PCA emerges a strong relation among DIM, BCS, and milk production. Therefore, with the increase in DIM, milk production increases and BCS increases as well. Moreover, THI, THI > 70, and illuminance are also correlated because when high values of illuminance are found, it can be assumed that the daylight is long (i.e., spring and summer) and the THI results in higher values as well. With regard to bouts, the number of bouts and their duration are set at opposite sides of the graph, meaning that, as mentioned above, a high number of bouts corresponds to their low duration, and vice versa.

With regard to lying time, this parameter is positioned in Figure 1, close to milk production, which indicates that high lying time helps increase milk production. Instead, lying time is far from THI on both axes; hence, at higher THI, lying time decrease. DIM and THI are both positioned far from the lying time, which means that high DIM and high THI negatively affect the duration of lying time. In PCA, LEI and BNEI were not included because they are calculated from milk production, lying time, and number of bouts per day, which are already included in the analysis. If included, their presence in PCA may drive the other parameters toward these two indexes.

Table 2 reports the factor patterns that define the above-mentioned relations quantified by means of FA. A proposed definition of the factor patterns affecting the results is as follows:

- Factor 1 = “Lactation and seasonality” is influenced by the increase in milk, DIM, BCS, illuminance, and THI (all factor patterns are positive).
- Factor 2 = “Behavior” is influenced by milk production, lying bouts, and the cows' willingness to lie down (the duration of bouts is the only negative factor pattern, which indicates that this parameter has an opposite trend with respect to the other affecting parameters).
- Factor 3 = “Weather” is affected by the meteorological variables of wind and rainfall (all factor patterns are positive).
- Factor 4 = “Lying and weather” is influenced by the lying time of cows and the meteorological variables (lying is the only negative factor pattern).

**Table 2:** Parameters and factor patterns resulting from FA.

**Parameters**	**Factor 1**	**Factor 2**	**Factor 3**	**Factor 4**
DIM	**0.729**	0.144	0.290	−0.215
Milk production	**0.405**	**0.467**	0.071	−0.050
Lying/day	0.067	**0.555**	0.348	**−0.530**
No. of bouts/day	−0.337	**0.828**	−0.234	0.101
Duration bouts/day	0.255	**−0.682**	0.403	−0.398
Illuminance	**0.664**	−0.113	−0.361	−0.005
THI	**0.819**	−0.064	−0.206	0.256
THI > 70	**0.687**	0.010	−0.299	0.316
Wind speed	0.187	0.020	**0.549**	**0.456**
Rainfall	−0.127	0.084	**0.616**	**0.584**
BCS	**0.644**	0.386	0.226	−0.135
Eigenvalues	2.93	1.87	1.42	1.23
Proportion	26.6	17.0	12.9	11.2

*Scores with values close to or higher than 0.5 are reported in bold*.

All of these factors support the previous findings and explain on which aspects to focus when studying the behavior of lactating cows.

In order to understand if a model can be developed based on these relations, the GLMSelect procedure was used. GLMSelect was calculated for lying time, as well as for LEI and BNEI. Together with lying time, LEI and BNEI were considered at this stage because, considering how they are calculated, they represent the relation between milk production and behavior. Therefore, identifying a model for these two indexes may achieve better and/or interesting results about the cows' behavior.

The results of all three models were statistically significant (*P* < 0.001), even if with a quite small *r*^2^ (0.15 for lying time, 0.29 for LEI, and 0.30 for BNEI). The sources with a fixed effect are the average standing time in the first 21 DIM (“standing time”), BCS at calving, milk production, and lactation stage. The sources with a covariate effect are the meteorological ones of illuminance, THI, and rainfall. Table 3 reports the results of the three models built on lying time, LEI, and BNEI.

**Table 3:** Model estimates for lying time, LEI, and BNEI resulting from the GLMSelect procedure.

Parameter^*^	**Lying**		**LEI**		**BNEI**	
	**Estimate**	**SE**	**Estimate**	**SE**	**Estimate**	**SE**
Intercept	13.90	0.38	1.32	0.13	0.39	0.26
Low standing time	1.16	0.09	−0.39	0.03	−1.14	0.06
Low BCS at calving	−0.22	0.09	−0.18	0.03	0.41	0.06
High milk production	0.05	0.09	0.70	0.03	0.87	0.06
Lact. stage 1	−1.72	0.12	0.12	0.04	−0.43	0.08
Lact. stage 2	−0.20	0.11	0.05	0.04	−0.16	0.08
Illuminance	−0.00042	0.00009	0.00011	0.00003	0.00041	0.00006
THI	−0.0476	0.0066	0.0211	0.0021	0.0562	0.0045
Rainfall	−0.0135	0.0056	0.0053	0.0018	0.0067	0.0038

*In every model are reported the estimate coefficient and the standard error (SE)*.

* *The parameters “high standing time,” “high BCS at calving,” “low milk production,” and “Lact. stage 3” are not in the table because their estimate values are 0*.

The most important effect on lying time results from the standing time and the lactation period. In the group “low standing time” (cows with standing time <14 h/day at 21 DIM), the model for the lying time shows that cows lie down for a longer time. In other words, cows lie down 1.16 h/day more above the intercept value if they were in the “low standing time” group at the beginning of the lactation period. When cows are in the first 50 DIM (Lact. stage 1), the lying time reduces by 1.72 h/day with respect to the intercept. Instead, when DIM increases, the effect on lying time decreases. Cows in the group “low BCS” (BCS at calving <3.25) had a lying time that is 0.22 h/day below the intercept for the whole lactation period.

The effect of the weather parameters is very restrained, especially for illuminance; every point of increase of THI and rainfall brings a slight decrease to the lying time, similar to what occurs on LEI and BNEI. For them, the standing time in the 21 DIM negatively affects both LEI and BNEI, meaning that an increase in the standing time at the beginning of the lactation period causes a reduction in the milk produced per hour of lying and per lying bout per day for the whole lactation period. Therefore, maintaining good management practices and adequate animal welfare at the beginning of the lactation period is important for the whole lactation period. Because LEI and BNEI are built on milk production, the group of high milk production influences the two indexes; in particular, with increases in milk production in the group of “high milk production” (>28 kg/day), LEI increases by 0.70 kg/h and BNEI increases by 0.87 kg/bout. BCS at calving and milk production in Lact. stage 1 (<50 DIM) shows a similar trend: with increasing values of BCS at calving, LEI decreases by 0.18 kg/h, while BNEI increases by 0.41 kg/bout; instead, with increasing values of milk production in Lact. stage 1 (<50 DIM), LEI increases by 0.12 kg/h and BNEI decreases by 0.43 kg/bout.

A further step in the statistical analysis is carried out using the logistic regression procedure. LA was done using a binary logit and Fishers' scoring as an optimization technique. Table 4 reports results of the LA with the model and the likelihood of incurring in the case of “low lying <11 h/day.” When the point estimate is >1, it is more probable to have “low lying,” while if the point estimate is <1, it is more probable to have “high lying” (>11 h/day). In fact, to increases in milk production level corresponds a higher likelihood of having a high lying time, which means that it is more probable to get a high lying time (>11 h/day) when cows are highly productive. Instead, it is 4.46 times more probable to get a low lying time when cows are at the beginning of the lactation period (Lact. stage 1, <50 DIM) with respect to later stages (Lact. stage 3, >100 DIM).

**Table 4:** Odds ratio estimates for the model of lying time and likelihood of incurring in a “low lying time” category.

**Effect**	**Point estimate**	**95% Wald confidence limits**	
Milk production high vs. low	0.87	0.72	1.05
Lact. stage 1 vs. >3	4.46	3.49	5.69
Lact. stage 2 vs. 3	1.14	0.93	1.41
Illuminance	1.00	1.00	1.00
THI	1.06	1.04	1.08
THI > 70	1.00	0.99	1.02
Rainfall	1.01	1.00	1.02

The weather effects are found to have a reduced influence on lying time; therefore, they marginally affect the probability of having a low lying time if these weather effects change. In particular, illuminance and THI > 70 had no effect.

**Figure 2:**
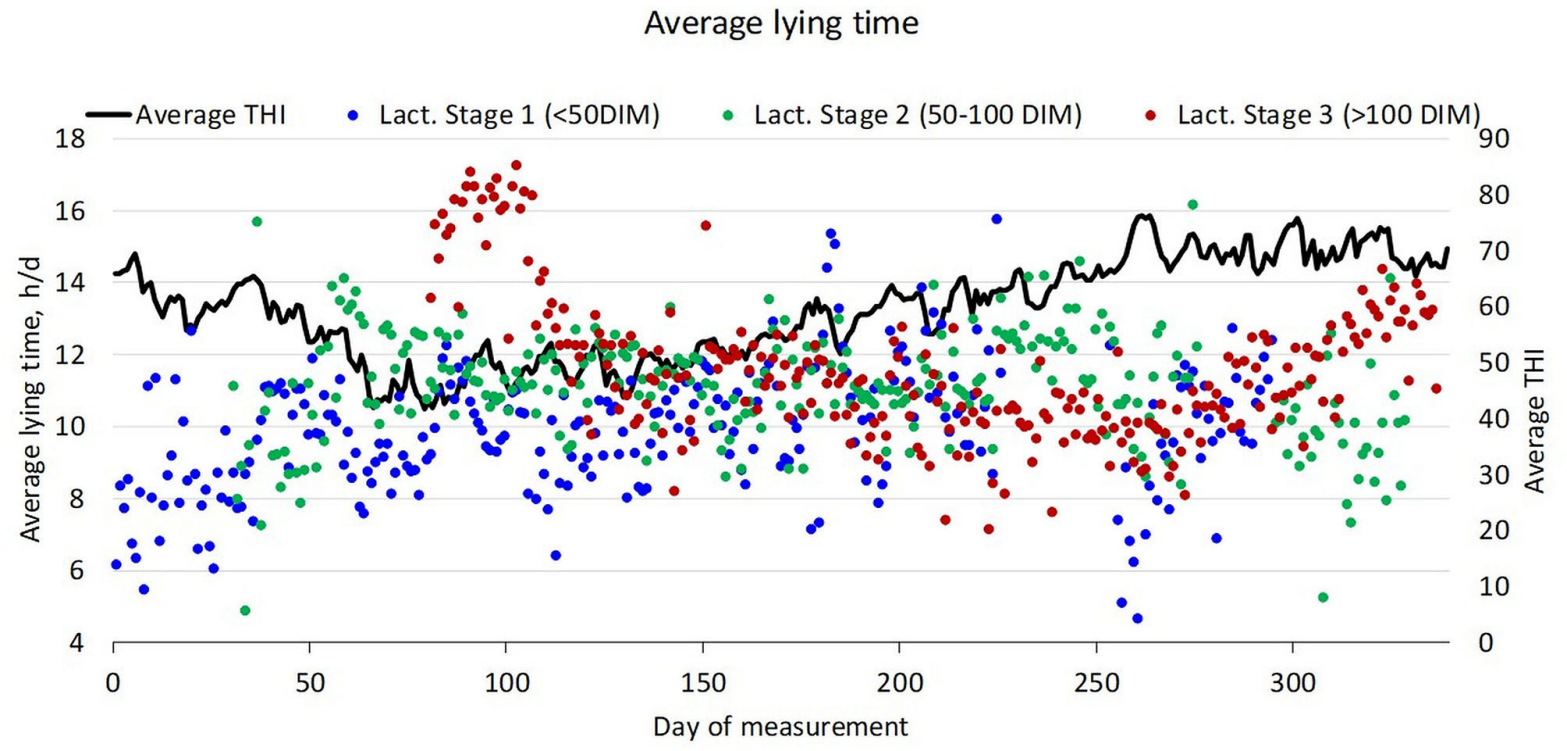
Trend along the year of the average lying time per day of cows in the three different lactation stages. The black continuous line represents the daily average THI value.

Given the importance emerging from the lactation stage, Figure 2 reports the trend over time of the lying time by taking into account the three lactation stages (Lact. stage 1, Lact. stage 2, and Lact. stage 3).

The black continuous line represents the daily average THI during the year. As the measurement started in September and lasted 1 year, THI is higher at the beginning and in the second part of the measurement period (i.e., spring and summer). THI in the three lactation stages was on average similar: THI ranged on average from 57.0 ± 8.3 in Lact. stage 1 to 60.2 ± 9.0 in Lact. stage 3. With respect to the lying time, although not statistically significant, cows in Lact. stage 1 (<50 DIM) show a lower average lying time (9.92 ± 1.72 h/day) than cows in the other stages. Lact. stage 3 shows instead a different trend; in fact, cows in this lactation stage highlight an average higher lying time with one peak in late autumn and a second trend of increase around the end of the monitored year (summer). For cows in Lact. stage 3 (>100 DIM), the average lying time was 11.61 ± 2.00 h/day. For cows in Lact. stage 2 (50–100 DIM), the average lying time was 11.11 ± 1.52 h/day.

Finally, through a cluster analysis, the farm observations were assessed and clustered as reported in Table 5. Four major clusters were identified.

**Table 5:** Mean of main parameters divided by cluster.

**Parameters**	**Clusters**				**SE**
	**A**	**B**	**C**	**D**	
No. of observations	889	316	895	375	
Milk production	28.7	28.3	26.5	29.4	0.13
DIM	87.8	89.1	65.7	95.4	0.89
BCS at calving	3.39	3.47	3.29	3.5	0.01
Lying time	10.9	10.8	11.0	10.6	0.05
LEI	2.74	2.75	2.50	2.92	0.02
BNEI	3.74	4.22	3.15	4.20	0.03
Illuminance	743	1,716	159	1,395	11.8
THI	59.3	59.5	51.26	62.93	0.18
THI > 70	7.82	4.61	0.18	10.21	0.21
Rainfall	2.01	0.61	8.16	2.73	0.17

Results are divided homogeneously in the four clusters, of which clusters A and C are the most numerous. Cluster C is characterized by the lowest milk production, lowest DIM, lowest BCS, highest lying time, lowest LEI and BNEI, lowest illuminance and THI, and highest rainfall. Therefore, this cluster includes the observations that lie in the winter period and that have calved shortly before (low DIM, low milk production, and low BCS).

D is the cluster in which cows with the highest milk production, the highest DIM (i.e., they are closer to the lactation curve's peak than cows in the other clusters), the highest BCS, the lowest lying time, the highest LEI and BNEI, and the highest THI and especially THI>70 are included. Together with high illuminance, this cluster is representative for those observations occurring in summer (highest THI and THI>70) and for those in which the primiparous cows are most productive. Clusters A and B showed intermediate means between clusters C and D, with cluster B being better than A with respect to LEI and BNEI. Illuminance is the highest in cluster B, which is therefore representative of the late spring period; in fact, in this cluster, THI and THI > 70 are also relatively high. In cluster B, rainfall is the lowest. Given that clusters A and B are very similar for milk production, DIM, and lying time, it seems that in cluster A are included cows that are in a more suffering condition. In fact, although THI is similar, THI > 70 is about 40% higher in cluster A than in cluster B. Since illuminance is much lower in cluster A than in cluster B but DIM is close in both groups, weather conditions (e.g., probably cloudy days) or defined areas of the barn may be responsible for such differences. Another option is that some cows suffer more than other cows, for example, because of a possible longer lying time in a part of the barn less adequate to the cows' welfare.

## Discussion

This study was characterized by monitoring primiparous dairy cattle for 1 year. From this research emerged findings that several aspects affect the behavior and in particular the lying time of cows. Environmental and productive aspects affect their behavior with interactions among each other.

The average milk production was recorded equal to 27.9 ± 6.5 kg/day, and cows lie down 10.6 ± 2.6 h/day, which is a quite low value compared to literature suggestions but still included within the range of adequate daily lying time. For example, Tullo et al. (13) found that cows commonly lie down for about 9–14 h/day when reared indoors, while Lovarelli et al. (14) showed that seasonality affects the lying time, in particular that the monitored cows lie down for a longer period in the cold season (on average 12.06 h/day) than in summer (on average 10.04 h/day). This supports also the findings in which a lower lying time is related to high THI conditions and in which high THI is associated with lower feed intake, lower milk production, and variations in behavior (9, 11, 17, 25, 26). Such a result was also obtained in this study, where it was found that one of the factors affecting lying time is seasonality and where the lactation stage (DIM), illuminance, THI, BCS, and milk production are the most relevant factors.

Although LEI and BNEI are quite simple indexes, they were found to give a very interesting indication on the importance of lying time and lying bouts. Every hour in which cows lie, they were found producing on average 2.68 ± 0.83 kg of milk. Avoiding those conditions that cause a reduction of lying time (e.g., heat stress) can therefore help increase milk production at more efficient levels. In particular, as mentioned, milk production was found to be affected by several variables among which are BCS at calving and daily lying time. Therefore, the management conditions that support optimal values of BCS at calving and lying time should be investigated and adopted adequately on the farm. Among them, for example, those reducing the negative effects caused by heat stress can play an important role. Natural and/or forced ventilation and shaded areas could improve the dairy cattle response to heat stress conditions that generally occur in summer (14, 27, 28). Specific feeding solutions with different frequency in feeding could also contribute in varying cow behavior (29). Indeed, THI has a very important effect on dairy cattle; as shown by Habeed et al. (15), every point of increase in THI > 69, a decrease of 0.41 kg/day per cow in milk production was found.

A very interesting result is obtained by LA that shows the likelihood of incurring in low lying time in defined conditions. In particular, it shows that it is more probable to have primiparous cows lying <11 h/day when the milk production level was low and especially when cows were in the first lactation stage (Lact. stage 1, at <50 DIM) with respect to when they were in the third lactation stage (Lact. stage 3, at >100 DIM). This means that it is very important to focus on the animals' welfare at the beginning of the lactation period and for its whole duration. In support of this, it was found that characteristics such as the standing time in the first 21 DIM affect the lying behavior during the whole lactation period. Finally, although cluster analysis results show that the most productive cluster is the one with the most reduced lying time, the differences among the clusters on this aspect are very small. Instead, it seems that clusters are mostly connected with the barn environment and weather conditions and that some cows suffer more than other cows from undesired environmental conditions (cluster A vs. cluster B, for example).

The evaluation of animals' behavior has positive applications on farms, as behavior is an important indicator of health and welfare. If this is guaranteed, farmers increase their possibilities in reducing production losses, as well as in health and welfare problems that derive from undesired climatic conditions in the barn. Moreover, the use of sensors for continuous real-time monitoring helps reduce the time-consuming human observation from the farmer (30–32).

Animals' welfare is known to be a very important aspect when dealing with livestock systems, because the living conditions of animals influence their productive performance (33, 34). For dairy cows, good performance refers to acceptable milk production. If welfare is maintained at adequate levels, then animals live in reasonable conditions that permit reaching satisfactory milk production levels. This can be connected with the topic of sustainable milk production, including the aspects related to environmental, economic, and social sustainability. According to this, environmental sustainability involves producing milk with an efficient balance between inputs used and outputs produced, as well as introducing inputs that have less impact on the environment with regard to categories such as acidification, eutrophication, and land use (35–37), that use renewable resources (38, 39), and that valorize at best the manure and slurry produced (40–42). Similarly, economic sustainability involves producing milk with the efficient use of inputs in such a way that the outputs permit achieving an economic profit. The easiest economic assessment for milk production involves using the income-over-feed cost (IOFC) parameter, which is widely adopted in literature (43, 44). However, studies in which a proper economic assessment is carried out are lacking, mostly because of farm-specific inputs and expenses. As regards social sustainability, this aspect is aimed at analyzing production, taking into account the welfare and health conditions of reared animals and of workers, which must be kept in a safe environment and work conditions (45, 46). Only recently has attention been paid to this aspect; therefore, there is still much to be done (45). In any case, the role of PLF can improve this sustainability aspect as well as the previously mentioned ones. In fact, using sensors that allow monitoring of the reared animals and installing tools that support the farmer and worker activity represent the first step toward guaranteeing better work conditions for farmers and workers. Moreover, monitoring animals allows identifying in real time possible problems in big herds, and therefore, the farmer can decide to intervene rapidly and only on the animals presenting health, welfare, reproductive, or productive problems (5, 31, 47–49).

## Conclusions

In this study, a sample of primiparous dairy cows was analyzed, and relationships among daily lying time, BCS at calving, milk production, and weather parameters were found. Two indexes, LEI and BNEI, were developed to evaluate milk production as a function of the animals' behavior (i.e., lying time and lying bouts), from which interesting results were found. The multivariate statistical analysis helped improve knowledge on the cattle sample. In fact, the complexity of the cattle farming system makes it difficult to exactly identify the influence of every parameter on each other, because cows respond differently to different conditions, for example, the surrounding environment. The lying time of dairy cows was found to be closely related to milk production, as widely found in literature. However, an interesting finding was that highly productive cows are more likely to have a high lying time (>11 h/day) and that cows at the beginning of the lactation period are more likely to have a low lying time. Moreover, the behavior in the initial lactation period affected the whole lactation. Monitoring single animals through sensors and IoT technology in accordance with PLF principles is becoming more important day by day.

## Data Availability Statement

The raw data supporting the conclusions of this article will be made available by the authors, without undue reservation.

## Ethics Statement

Ethical review and approval was not required for the animal study because no analysis was done on animals but only an assessment on their behavior.

## Consent to Publication

The monitored livestock farm is a commercial farm whose owner is the University of Milan; therefore, the University of Milan owns the data, and the authors have the consent to use them.

## Author Contributions

DL, AT, and GP: conceptualization and methodology. DL and AT: formal analysis and writing—revision preparation. GM, MZ, and ER: investigation. DL, AT, GM, and MZ: data curation. DL, AT, ER, and GP: writing—original draft preparation. GP and MG: supervision. MG: project administration and funding acquisition. All authors have read and agreed to the published version of the manuscript.

## Conflict of Interest

The authors declare that the research was conducted in the absence of any commercial or financial relationships that could be construed as a potential conflict of interest.
